# Assessing the Complex and Evolving Relationship between Charges and Payments in US Hospitals: 1996 – 2012

**DOI:** 10.1371/journal.pone.0157912

**Published:** 2016-07-08

**Authors:** Hannah Hamavid, Maxwell Birger, Anne G. Bulchis, Liya Lomsadze, Jonathan Joseph, Ranju Baral, Anthony L. Bui, Cody Horst, Elizabeth Johnson, Joseph L. Dieleman

**Affiliations:** 1 Institute for Health Metrics and Evaluation, University of Washington, Seattle, Washington, United States of America; 2 Global Health Group, University of California San Francisco, San Francisco, United States of America; 3 Northwell Health, Great Neck, New York, United States of America; 4 David Geffen School of Medicine, University of California Los Angeles, Los Angeles, United States of America; UNAIDS, GUYANA

## Abstract

**Background:**

In 2013 the United States spent $2.9 trillion on health care, more than in any previous year. Much of the debate around slowing health care spending growth focuses on the complicated pricing system for services. Our investigation contributes to knowledge of health care spending by assessing the relationship between charges and payments in the inpatient hospital setting. In the US, charges and payments differ because of a complex set of incentives that connect health care providers and funders. Our methodology can also be applied to adjust charge data to reflect actual spending.

**Methods:**

We extracted cause of health care encounter (cause), primary payer (payer), charge, and payment information for 50,172 inpatient hospital stays from 1996 through 2012. We used linear regression to assess the relationship between charges and payments, stratified by payer, year, and cause. We applied our estimates to a large, nationally representative hospital charge sample to estimate payments.

**Results:**

The average amount paid per $1 charged varies significantly across three dimensions: payer, year, and cause. Among the 10 largest causes of health care spending, average payments range from 23 to 55 cents per dollar charged. Over time, the amount paid per dollar charged is decreasing for those with private or public insurance, signifying that inpatient charges are increasing faster than the amount insurers pay. Conversely, the amount paid by out-of-pocket payers per dollar charged is increasing over time for several causes. Applying our estimates to a nationally representative hospital charge sample generates payment estimates which align with the official US estimates of inpatient spending.

**Conclusions:**

The amount paid per $1 charged fluctuates significantly depending on the cause of a health care encounter and the primary payer. In addition, the amount paid per charge is changing over time. Transparent accounting of hospital spending requires a detailed assessment of the substantial and growing gap between charges and payments. Understanding what is driving this divergence and generating accurate spending estimates can inform efforts to contain health care spending.

## Background

In 2013 the United States spent $2.9 trillion on health care, more than in any previous year [1]. In an effort to bend the health care cost curve and to improve quality and transparency, the Affordable Care Act included a series of payment reforms which substantially affect hospitals [2]. Much of the debate around hospitals and cost containment is centered on the complicated and often disjointed pricing system for hospital services [3,4]. To address the persistent rise in US health care spending, it is essential to grasp the nuances of hospital spending. Our investigation contributes to the health care spending knowledge base by assessing the relationship between payments and charges in the inpatient setting.

Up until the 1980s, inpatient charges mirrored payments closely and were a valid proxy for spending in the US. However, in recent years charges have increased dramatically and payments have not kept pace [5]. This divergence likely stems from a complex interplay of incentives that exist in today’s US health care system. Payments to hospitals are negotiated based on varying criteria, which may involve external estimates of the cost of service provision, or a fixed percentage of billed charges [4]. The specifics depend on the insurer and are periodically altered [6,7]. Hospitals may respond to changes in payment rates in a number of ways, including increasing operating efficiency, but also by providing differential services to patients depending on their insurance, attempting to attract patients with a specific make-up of insurance coverage, or cutting financial assistance programs [4,6,7]. They may also alter their charging policy to maximize revenue, rather than tying charges to the cost of providing a given service. In this case, the amount charged for a given good or service is often non-intuitive. Moreover, this phenomenon can place the most burden on those with the least bargaining power, as the uninsured are often confronted with the largest bills [4]. These charges may have little to do with the cost of the services received. This widening gap means that charge data are increasingly insufficient for estimating how much was actually paid.

We approach health care spending from the perspective of the payer. In this framework, spending on inpatient hospital care should be measured by tracking payments to hospitals, because payments are the amount of money actually spent by patients and insurers for care. Unfortunately, data on inpatient payments are often not easily accessible. In contrast to payment data, information on inpatient charges is easier to obtain. With an understanding of the ratio between charges and payments, we are able to adjust these comparatively ample charge data to reflect payments. In this study, we model the payment-to-charge ratio (payment ratio) in the inpatient setting from 1996 to 2012 by cause of health care encounter (cause) and primary payer type (payer)–private insurance, public insurance, and out-of-pocket payments. We then apply these payment ratios to a high quality dataset that contains only charge data in order to produce cause-specific payment estimates over time.

We had two primary objectives for conducting this research. First, since charges do not vary by payer, analyzing the relationship between payments and charges enables us to understand who bears the burden of financing US hospital health care, and there is limited research exploring this relationship [8]. We generated nationally representative hospital inpatient payment ratios across time and stratified by payer and cause. Using payment and charge data from the Medical Expenditure Panel Survey (MEPS), we modeled a cause- and payer-specific linear relationship between the payment ratio and time. Exploring the payment ratio by cause is a natural approach for several reasons. Medicare determines payments based heavily on Medicare Severity Diagnosis Related Groups (MS-DRGs), which are approximated by the cause groupings used in this study [9]. While the exact correspondence varies, private payers generally follow Medicare’s lead in determining reimbursements [7,10,11]. In addition, inspection of the data reveals that cause is related to the payment ratio, as detailed below and in the S1 File. A thorough understanding of the trends and characteristics of the payment ratio as it relates to time, payer, and cause can help illuminate market malfunctions and thereby inform health policy to contain costs and improve health care quality.

Second, we were motivated to develop payment ratios in the inpatient setting as a methodological tool for the broader goal of tracking spending on health. Most health care spending research tracks actual payments (also known as spending) [12–14]. However, charge data, such as the National Inpatient Sample (NIS), are often more readily available than payment data. Developing cause-, payer-, and year-specific payment ratios allows researchers to adjust charges to reflect payments at a granular level, and thereby render data sources with only charge data usable for estimating spending.

## Methods

In this paper we estimated payment ratios for inpatient health care in the United States. We regressed the ratio of a patient’s total payments over their total charges on a time trend and indicators identifying characteristics of the inpatient stay. We used these findings to obtain payment estimates from the National Inpatient Sample (NIS) dataset, which reports charges only.

### Data and data pre-processing

To estimate payment ratios in the hospital inpatient setting, we used nationally representative data tracking inpatient stays between 1996 and 2012 from the Medical Expenditure Panel Survey (MEPS). MEPS is produced annually by the US Agency for Health care Research and Quality (AHRQ) and provides estimates on spending and health care utilization in the non-institutionalized civilian population. Household underreporting and underrepresentation of people with high spending is known to lead to downward-biased spending estimates in MEPS [5]. However, because it covers many years and is nationally representative, MEPS is still a valuable and frequently used dataset for studying health care spending. In addition, MEPS reports both charges and payments for each inpatient stay, and it captures health encounters by the uninsured and those paying primarily out-of-pocket, making it an ideal input dataset for our model. We obtained diagnoses, facility charges, facility payments, doctor charges, doctor payments, age, sex, year, and patient weights from MEPS.

We classified patients in five-year age groups extending to an open category of those older than 85 years. We split the 0–4 age group into a 0–1 bin and a 1–4 bin because of unique health care provided during the first year of life. We took the first listed diagnosis to be the primary diagnosis. We categorized payments by three types of payers: private, public, and out-of-pocket. Private insurance is insurance that is not administered by the government, with plans often provided through employers but sometimes purchased separately. Public insurance consists of government programs including Medicaid and Medicare, which insure those who qualify based on factors like income or age. Out-of-pocket payments come directly from individuals, rather than through insurance. Many inpatient stays reported payments from multiple payers, reflecting co-payments, deductibles, or the purchase of Medicare Supplement Insurance. When multiple payers were listed, we designated the payer who covered the largest portion of the payment as the primary payer. Payments categorized as out-of-pocket could include both patients without insurance and those with high-deductible plans. MEPS lists diagnoses using the International Classification of Disease version 9 (ICD-9) system [15]. We used the Global Burden of Disease 2013 study (GBD) as our underlying framework for disease classification [16]. This cause framework aggregates the 17,849 ICD-9 codes into 289 distinct causes based on clinically relevant groupings of codes [17]. Within the GBD framework, causes can be further aggregated into less granular classifications depending on the policy purpose. In this study we also included an “expenditure-only” category of causes, which tracks encounters with the health system that are not associated with disease burden, and are therefore excluded from GBD. Examples are organ donation, and healthy pregnancy and postpartum care. We mapped the primary diagnoses found in MEPS to 32 unique causes of health encounters. MEPS truncates ICD-9 codes to three digits for privacy reasons. To map ICD-9 codes more exactly to GBD causes, we probabilistically replaced the existing ICD-9 codes with full, five-digit equivalents. More description of this replacement process is provided in the S1 File. MEPS has some observations with ICD-9 codes that do not map to valid GBD causes, such as “fracture of unspecified bones,” “certain early complications of trauma,” and “care involving use of rehabilitation procedures” [18]. Since the underlying cause of the health encounter was not clear, these observations were considered “garbage causes” and were excluded. Injury-related causes are sometimes coded to the “nature” of injury, such as a broken hip, and other times to the “external” cause of injury, such as a car accident. These latter types of injury codes are used by GBD, as they are deemed to be more policy relevant because they can inform prevention measures. In order to ensure that spending was attributed to these external injury codes, we probabilistically reassigned nature injury codes to external causes when only a nature code was provided [19]. More detail on these methods can be found in the S1 File.

Some observations in MEPS report larger total payments than total charges (2.2% of observations). Based off the assumption that a patient would not pay more than charged for a medical procedure, we considered these observations to be errors, and we reset these charges to be equal to payments. To test this assumption, we ran a sensitivity analysis by keeping these charges as they were. This alteration did not qualitatively change our results. Details of this additional analysis are reported in the S1 File.

Due to relatively small sample size and the possibility of missing rare causes of health encounters, a moving average approach was used for MEPS, such that data from each year were added to the annual estimates for that year as well as the adjacent years before and after. As a consequence, our 1997 estimates include data from 1996 and 1998, and trends over time are measured to be more gradual. This approach is similar to what is recommended by AHRQ and methods used by Dunn and colleagues at the US Bureau of Economic Analysis [20].

### Statistical methods

We ran cause-specific linear regressions in which payment ratios for an inpatient encounter (a single stay in an inpatient facility) are a function of payer and year. We imposed a linear time trend, but allowed the time trends to be distinct by payer. Inspecting patient-level ratios showed that these payment ratios did not vary systematically by age or sex, so we did not include these as additional controls. For each cause, the estimated linear equation was:

$${\left(\frac{payments}{charges}\right)}_{i}=\;\beta_{0}\cdot public_{i}+\;\beta_{1}\cdot private_{i}+\;\;\beta_{2}\cdot oop_{i}+\;\beta_{3}\cdot public_{i}\cdot year+\;\beta_{4}\cdot private_{i}\cdot year+\;\beta_{5}\cdot oop_{i}\cdot year+\;\varepsilon_{i}$$

We generated regression weights for each observation by taking the product of the total charge and the patient weight provided by MEPS. These custom weights allowed our regression to be appropriately adjusted for two issues. First, the patient weights from MEPS addressed the survey’s sampling frame. Using these patient weights made our results representative of the demographics of the civilian non-institutionalized US population. Second, incorporating charges into the regression weights allowed us to up-weight larger charges, making our estimates representative of every dollar charged.

We applied the above regression for each of the 32 causes that had more than 200 observations between 1996 and 2012. This threshold was met for 58% of the iterations of the analysis. Out of the instances for which the threshold was not met, 48% were for out-of-pocket payers. When this threshold was not met, we included a single year trend, rather than a payer-specific year trends.

### Sensitivity analyses and uncertainty

In order to provide a complete set of estimates, MEPS inpatient data are provided with missing payment and charge data imputed. If measurement error from AHRQ’s imputation is nonrandom, this adjustment could bias our estimates. To assess this possibility we completed a sensitivity analysis in which we weighted our regression by an index identifying how much imputation was necessary to complete each observation’s record. This weighting system did not substantively alter our results. See S1 File for more detail and these results.

In this study we used a linear model to describe the relationship between payment ratios and time. To test the validity of assuming a linear relationship between payment ratio and time, we used a natural spline regression model to flexibly estimate a nonlinear change across time. Allowing for nonlinear time trends did not substantively alter our results. See S1 File for more detail and these results.

We bootstrapped all our data 1,000 times to generate uncertainty estimates. We ran all analyses 1,000 times and calculated 95% uncertainty intervals for all estimates using the bootstrap draws.

### Applying payment ratios to secondary charge data to track health spending

The NIS is a nationally representative sample that reports charges, but not actual payments. In order to convert NIS charge estimates to payment estimates, we applied the predicted payment ratios calculated from MEPS and stratified by payer, year, and cause to NIS.

Like MEPS, NIS is produced by AHRQ. It is the largest publicly available all-payer inpatient health care sample in the United States. With data from six to eight million inpatient hospital stays per year, NIS produces nationally representative estimates of health care utilization and facility charges in the inpatient setting [21]. We obtained ICD-9 codes for primary and secondary diagnoses, facility charges, age, sex, year, and patient weights from NIS. We used the same methodology to process NIS as we did to process MEPS. We binned ages, mapped ICD-9 codes to GBD causes, and adjusted injury-related observations.

To convert the nationally representative charge data in NIS into nationally representative payment estimates, we also needed to adjust NIS facility charges to reflect total charges. NIS facility charges exclude professional fees [18]. Using MEPS, we ran a similar regression to estimate the ratio between facility charges and total charges, stratified by cause and year. Details of this method appear in the S1 File.

**Fig 1:**
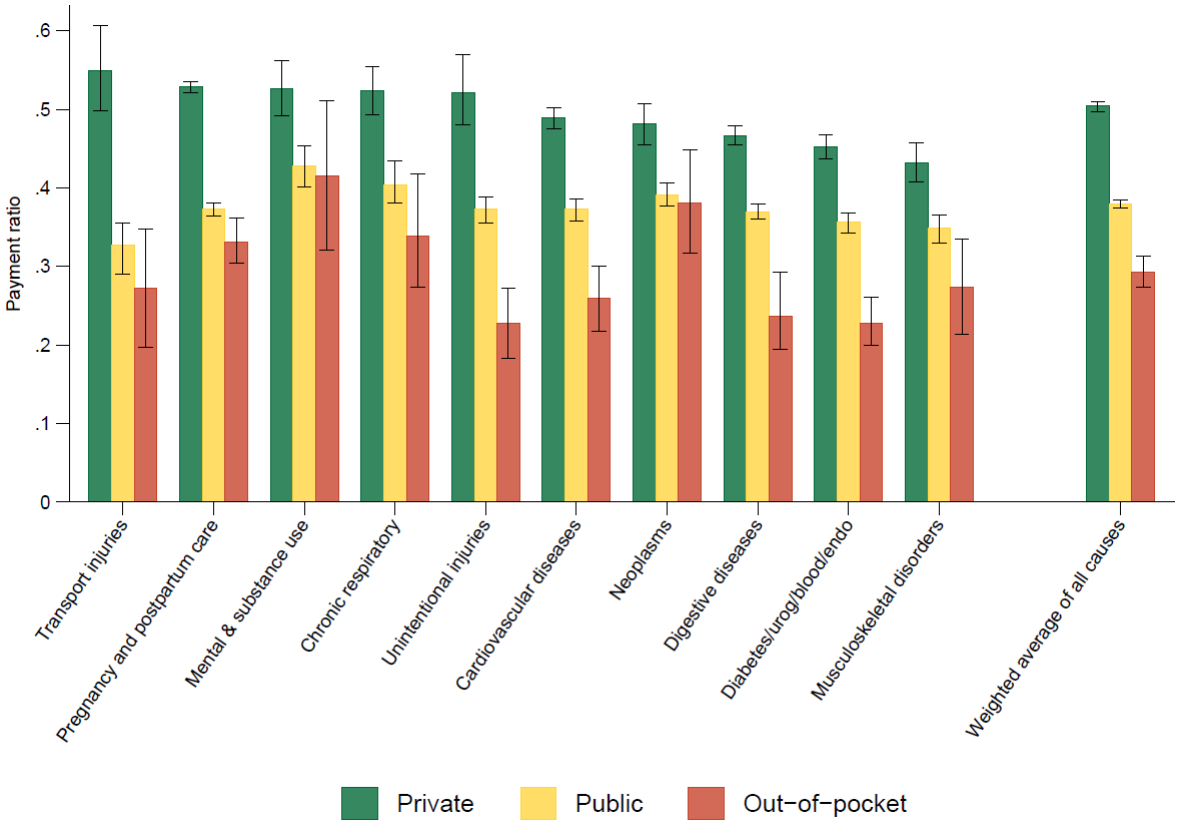
Weighted average from 1996–2012 of estimated payment ratios with 95% uncertainty intervals, shown by payer for the 10 largest causes of spending and the weighted average of all causes.

**Fig 2:**
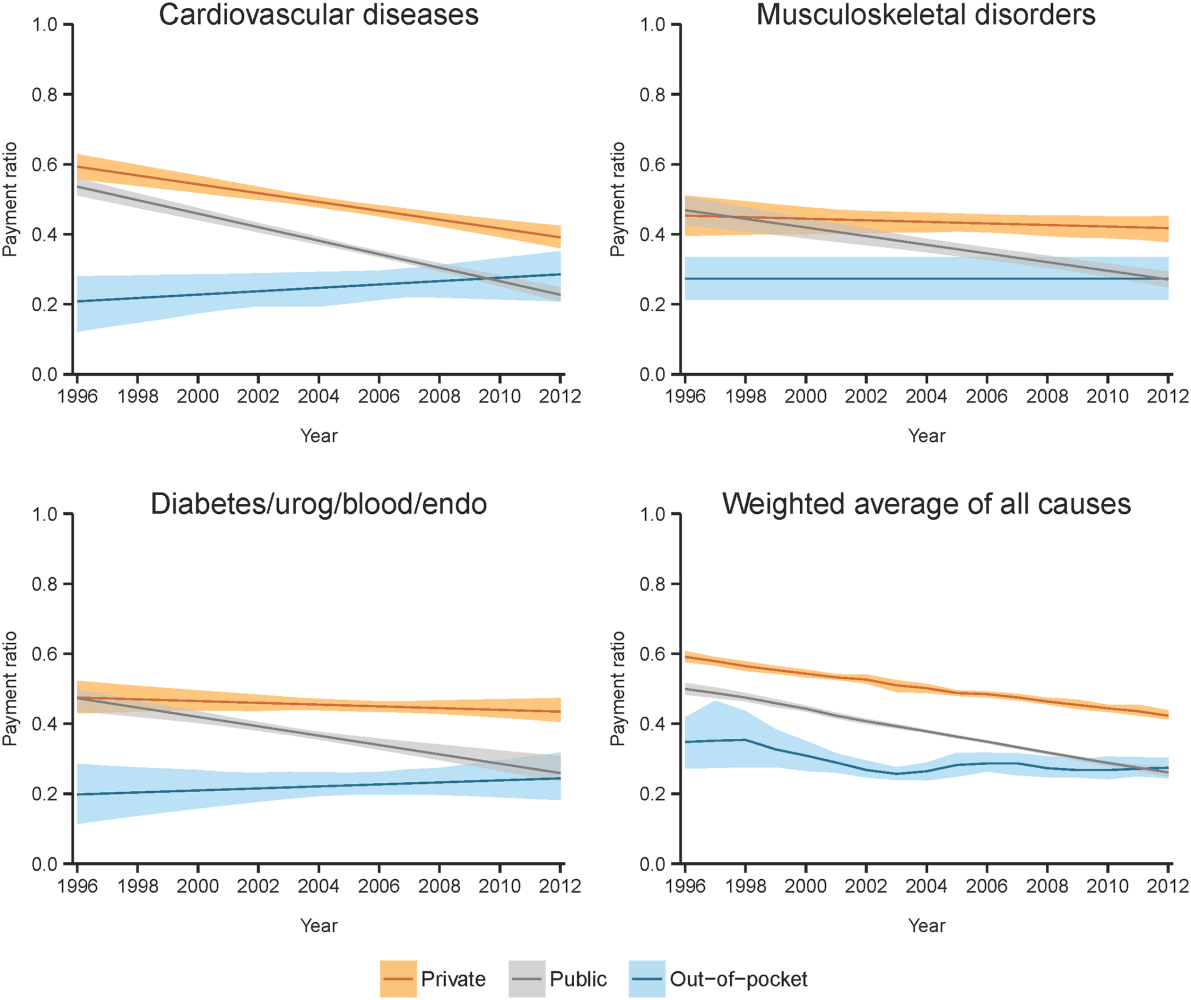
Time trends of estimated payment ratios with 95% uncertainty intervals, shown by payer for select causes and the weighted average of all causes.

**Fig 3:**
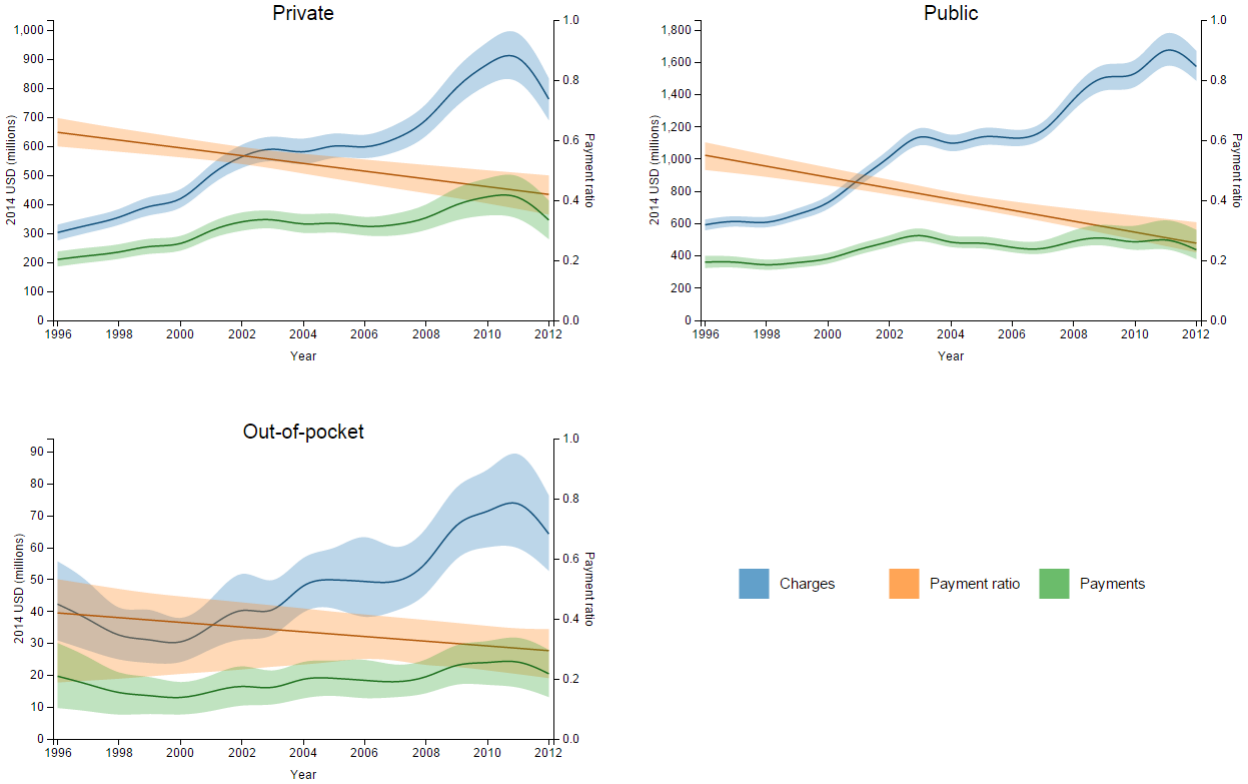
Interstitial lung disease charges and estimated payments in NIS, with estimated payment ratios and 95% uncertainty intervals, shown for each payer over time.

**Fig 4:**
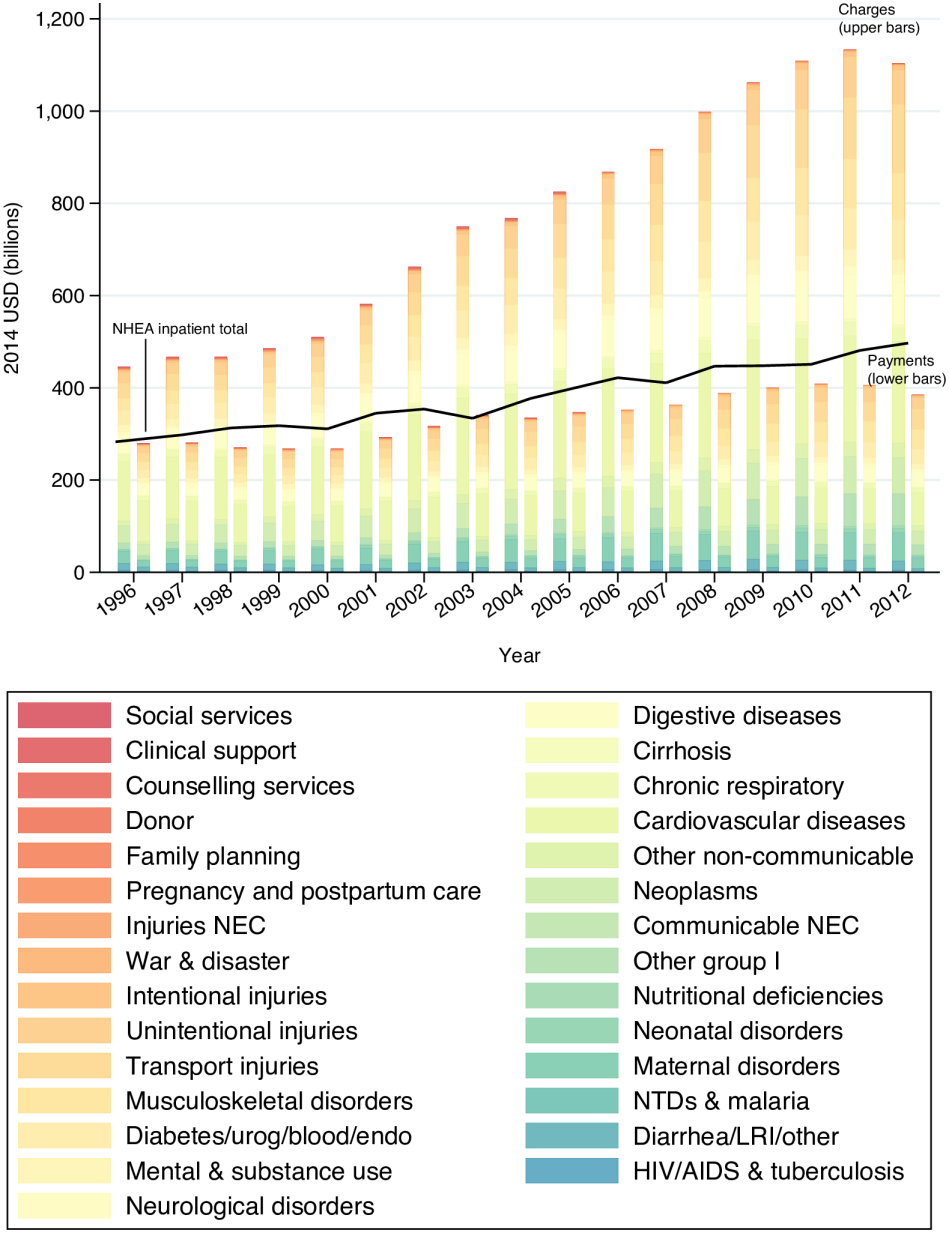
Time trends of all-payer charges and estimated payments in NIS, stratified by cause, with the NHEA inpatient total shown for comparison.

All statistical analyses were run in Stata13 [22]. Fig 1 was created using Stata13 [22]. Fig 2 was made using ggplot2 in R 3.0.0 [23,24].Fig 3 was done in D3 [25]. Fig 4 was created using Stata13 [22].

## Results

We used hospital inpatient data from MEPS for 1996–2012 to derive payment ratios constructed to reflect how much each payer paid on average per dollar charged. These estimates are stratified by primary payer, cause, and year. Our processed dataset included 197,263 inpatient hospital stays. This total is larger than the original number of observations in MEPS, due to the moving average and the draw-specific assignment of five-digit ICD-9 codes undertaken during processing. This expansion is explained in more detail in the S1 File. The primary payer for 59% of the stays was public insurance, 35% was private insurance, and 6% were patients who paid out-of-pocket. These represented 61%, 36%, and 3% of the charges, respectively. The 10 largest causes of inpatient health care spending as measured by our study were cardiovascular diseases; neoplasms; pregnancy and postpartum care; digestive diseases; diabetes, urogenital, blood, and endocrine diseases (DUBE); unintentional injuries; musculoskeletal disorders; chronic respiratory diseases; mental and substance use disorders; and transport injuries. In 1996, the average payment ratios across these 10 causes (weighted by total payment for each cause) were 0.59, 0.51, and 0.36, for private insurance, public insurance, and out-of-pocket, respectively. In 2012, the average payment ratios for these 10 causes were 0.41, 0.26, and 0.28.

Fig 1 illustrates the variation in average payment ratio by payer and cause. Within these 10 causes, the estimated payment ratio for private payers was consistently the largest, followed by public, and then out-of-pocket. The distinction between the three payers was statistically significant for all of these 10 causes (all p-values < 0.01). With payment ratios of 0.43 and 0.42, public insurers and those paying out-of-pocket paid more per dollar charged for mental and substance use disorder treatment than for any of the other 10 largest causes of spending. The highest payment ratio for private payers was transport injuries, with a value of 0.55. Between private and public payment ratios, the highest differential between payment ratios was 0.22 for transport injuries. The lowest differential was for musculoskeletal disorders, with a value of 0.07. In other words, when someone had an inpatient stay for transport injuries, private insurers paid 22 more cents per dollar charged than public insurers, whereas private insurers paid only 7 more cents per dollar charged when covering musculoskeletal disorders care. Between the private and out-of-pocket payment ratios, the highest differential was 0.32 for unintentional injuries, and the lowest was 0.10 for neoplasms. Between the public and out-of-pocket payment ratios, the greatest differential was for unintentional injuries and the lowest was for neoplasms, with values of 0.17 and 0.02, respectively.

To get a higher level view of the variation in payment ratios by payer, we generated a weighted average of payment ratios over all causes and years using payment data from MEPS as the weights. For this weighted average, the payer-specific payment ratios were as follows: 0.50 for private, 0.38 for public, and 0.29 for out-of-pocket.

Figs 2 and 3 show how the estimated payment ratios change over time for different payers within a cause. Fig 2 depicts the time trends for payment ratios for the three payers within a cause in order to facilitate comparison between payers. Fig 3 illustrates the relationship between charges and payments for a given payer and cause by showing these metrics on the same scale (left axis), overlaid by the corresponding payment ratio (right axis). Payment ratios tended to decline or remain constant for private and public payers, while for out-of-pocket there were several increasing trends. In the case of DUBE, the private and out-of-pocket payments relative to charges remained mostly stable but the public payment ratio declined over time. For unintentional injuries and digestive diseases, the payment ratios declined across all payers. For the remaining seven of the ten largest causes of spending, some payer-specific payment ratios crossed over the course of the time period. Table 1 details the numbers behind Fig 2, including the starting and ending payment ratios over the 16-year time period, as well as the direction of the time trend for each cause and payer combination.

**Table 1:** Estimated payment ratios by cause and payer in 1996 and 2012, with 95% uncertainty intervals and direction of change over time.

Cause	Private payers			Public payers			Out of pocket payers		
	1996	2012	Direction of change	1996	2012	Direction of change	1996	2012	Direction of change
All causes weighted	0.59 (0.61, 0.58)	0.42 (0.44, 0.41)	-	0.50 (0.52, 0.48)	0.26 (0.28, 0.25)	-	0.35 (0.42, 0.27)	0.27 (0.30, 0.24)	-
HIV/AIDS & tuberculosis	0.48 (0.59, 0.38)			0.48 (0.62, 0.35)	0.25 (0.34, 0.16)	-	0.08 (0.15, 0.03)		
Diarrhea/LRI/other	0.69 (0.76, 0.62)	0.41 (0.47, 0.34)	-	0.53 (0.58, 0.49)	0.27 (0.31, 0.24)	-	0.19 (0.53, 0.09)	0.12 (0.24, -0.12)	-
NTDs & malaria	0.52 (0.71, 0.36)			0.20 (1.00, 0.04)			1.00 (1.00, 1.00)		
Maternal disorders	0.50 (0.59, 0.42)	0.44 (0.56, 0.35)	-	0.43 (0.48, 0.37)	0.19 (0.24, 0.16)	-	0.17 (0.23, 0.12)		
Neonatal disorders	0.62 (0.73, 0.51)			0.28 (0.38, 0.22)	0.27 (0.30, 0.21)	-	1.00 (1.00, 1.00)		
Nutritional deficiencies	0.56 (0.65, 0.45)	0.56 (0.66, 0.46)	+	0.46 (0.53, 0.37)	0.25 (0.30, 0.20)	-	0.16 (0.25, 0.04)		
Other communicable, maternal, neonatal, and nutritional diseases	0.69 (0.81, 0.57)	0.51 (0.59, 0.41)	-	0.54 (0.59, 0.48)	0.23 (0.27, 0.20)	-	0.27 (0.40, 0.15)		
Communicable not elsewhere classified	0.63 (0.75, 0.52)			0.30 (0.50, 0.24)			0.12 (0.22, 0.04)		
Neoplasms	0.61 (0.65, 0.57)	0.34 (0.40, 0.31)	-	0.51 (0.54, 0.48)	0.28 (0.31, 0.25)	-	0.53 (0.66, 0.41)	0.22 (0.31, 0.12)	-
Other non-communicable	0.53 (0.58, 0.48)	0.55 (0.61, 0.49)	+	0.46 (0.51, 0.40)	0.25 (0.29, 0.22)	-	0.38 (0.49, 0.24)	0.26 (0.44, 0.16)	-
Cardiovascular diseases	0.59 (0.63, 0.56)	0.39 (0.42, 0.36)	-	0.54 (0.56, 0.51)	0.23 (0.25, 0.21)	-	0.21 (0.28, 0.12)	0.29 (0.35, 0.21)	+
Chronic respiratory	0.63 (0.67, 0.58)	0.42 (0.48, 0.35)	-	0.55 (0.59, 0.50)	0.26 (0.33, 0.23)	-	0.42 (0.53, 0.19)	0.29 (0.37, 0.20)	-
Cirrhosis	0.33 (0.40, 0.28)	0.34 (0.41, 0.29)	+	0.23 (0.53, 0.11)	0.31 (0.53, 0.21)	+	0.13 (0.26, 0.06)		
Digestive diseases	0.57 (0.60, 0.55)	0.38 (0.41, 0.35)	-	0.47 (0.49, 0.44)	0.28 (0.30, 0.26)	-	0.32 (0.42, 0.23)	0.18 (0.23, 0.12)	-
Neurological disorders	0.66 (0.71, 0.60)	0.38 (0.43, 0.33)	-	0.48 (0.53, 0.39)	0.24 (0.29, 0.21)	-	0.15 (0.20, 0.10)		
Mental & substance use	0.62 (0.70, 0.56)	0.41 (0.47, 0.36)	-	0.57 (0.60, 0.52)	0.28 (0.31, 0.26)	-	0.48 (0.63, 0.27)	0.30 (0.42, 0.19)	-
Diabetes/urog/blood/endo	0.47 (0.52, 0.43)	0.43 (0.47, 0.41)	-	0.47 (0.50, 0.44)	0.26 (0.31, 0.23)	-	0.20 (0.29, 0.11)	0.24 (0.32, 0.18)	+
Musculoskeletal disorders	0.45 (0.51, 0.40)	0.42 (0.45, 0.38)	-	0.47 (0.51, 0.43)	0.27 (0.29, 0.25)	-	0.27 (0.33, 0.21)		
Transport injuries	0.71 (0.80, 0.63)	0.41 (0.47, 0.36)	-	0.44 (0.53, 0.28)	0.24 (0.32, 0.19)	-	0.27 (0.35, 0.20)		
Unintentional injuries	0.62 (0.69, 0.55)	0.44 (0.50, 0.39)	-	0.49 (0.53, 0.44)	0.27 (0.29, 0.25)	-	0.35 (0.43, 0.25)	0.08 (0.15, 0.00)	-
Intentional injuries	0.60 (0.71, 0.44)	0.49 (0.58, 0.41)	-	0.43 (0.53, 0.35)	0.25 (0.30, 0.20)	-	0.20 (0.31, 0.12)		
War & disaster	0.45 (0.59, 0.35)			0.48 (0.55, 0.41)			0.24 (1.00, 0.11)		
Injuries not elsewhere classified	0.56 (0.68, 0.44)			0.35 (0.46, 0.27)			0.18 (0.67, 0.01)		
Pregnancy and postpartum care	0.61 (0.63, 0.60)	0.46 (0.47, 0.45)	-	0.45 (0.46, 0.43)	0.32 (0.33, 0.30)	-	0.21 (0.27, 0.15)	0.42 (0.47, 0.37)	+
Family planning	0.79 (1.00, 0.76)			0.29 (0.41, 0.22)			0.22 (1.00, 0.00)		
Donor	1.00 (1.00, 1.00)			1.00 (1.00, 1.00)			1.00 (1.00, 1.00)		
Counselling services	0.34 (0.42, 0.23)			0.34 (0.38, 0.31)			0.22 (0.41, 0.01)		
Clinical support	0.57 (0.71, 0.45)			0.31 (0.37, 0.26)			0.25 (1.00, 0.03)		
Social services	1.00 (1.00, 1.00)			0.89 (1.00, 0.40)			1.00 (1.00, 1.00)		

Finally, we applied the payment ratios to NIS charges to obtain nationally representative payment estimates. Fig 4 contrasts the time trends in charges and payments, stratified by cause. The reported charges from NIS are shown adjacent to the adjusted payment estimates in each year. The line running near the top of the payments bars represents a separate yearly estimate for inpatient care, which is derived from the National Health Expenditure Accounts (NHEA) estimate for hospital spending. To align the NHEA hospital spending with our definition of inpatient care, we applied an adjustment used in previous research and explained in more detail in the S1 File [14,26]. We subtracted out spending attributed to “garbage causes” from this NHEA estimate. The proximity of the NHEA estimate to the adjusted NIS estimates serves as an external validation of our methods. Further, the gap between these two estimates is readily explained by the fact that the NHEA estimate includes non-operating revenue, which is out of scope of NIS. Therefore, we expect our adjusted NIS spending estimates to be below the NHEA line [27]. The NIS adjusted spending estimates presented in this graph confirm this prior expectation.

While both charges and payments increased from 1996–2012, charges increased more rapidly, particularly accelerating in the early 2000s. In 1996, $445.7 billion was charged for inpatient health care for causes in our sample, increasing to $1.1 trillion in 2012. Of these charges, the amount paid increased much less, going from $279.6 billion in 1996 to $385.1 billion in 2012. The largest cause of both charges and payments in 2012 was cardiovascular diseases, with $221 billion charged and $70.9 billion paid. This was followed by transport injuries and musculoskeletal disorders, with payment amounts of $44.9 billion (30.1% of the charges) and $42.6 billion (42.2% of the charges), respectively.

## Discussion

Amidst the current effort to increase transparency in US health care, it is valuable to grasp the relationship between how much a patient is charged and how much is paid [2]. At a broad level, the finding that $1.1 trillion were charged for inpatient stays at hospitals in 2012 and only $385.1 billion were paid highlights the peculiarity of hospital pricing in the US and encourages a more transparent pricing system. Additionally, understanding how this relationship varies over time—both by type of primary payer, and by the cause of the health care encounter—can help researchers and policymakers evaluate the sustainability and equity of the system at a granular level. Examining the payment ratios can identify target areas for change, including by improving the cost-efficiency of certain treatments, minimizing overhead costs, altering payment policy to hospitals for a given DRG or insurance type, or keeping in check certain charging policies. The payment ratios also serve as a valuable tool for estimating payments when only charge data are available, which can lead to more accurate accounts of health care spending in the US. In turn, knowing about health care spending at a detailed level helps to connect health care finances with the burden of disease in the US, leading to better-informed policy decisions, and facilitating efforts to minimize spending overall.

Our study finds that payment ratios vary considerably across three dimensions. First, payment ratios have changed over time. We find that in 1996, an average of 54 cents were paid per dollar charged for an inpatient stay. By 2012, the average payment went down to only 34 cents per dollar charged. However, during this period, total inpatient spending has increased nearly 57%, from $437 billion in 1996 to $685 billion in 2012 [1,28]. Thus, inpatient charges have gone up even faster than inpatient payments. The expanding divergence of payments from charges highlights the unique and complex economic position of health care in the US, as hospitals, insurance companies, and government agencies struggle with seemingly conflicting priorities and capabilities.

Second, our study finds that payment ratios vary substantially by payer. Among the 10 largest causes of inpatient health care spending, public insurers paid an average of 38 cents per dollar charged during the period of our study, whereas private insurers and out-of-pocket payers paid 50 and 30 cents per dollar charged, respectively. For all of these 10 causes, out-of-pocket ratios were significantly lower than private and public ratios (p-value < 0.01), and public ratios were significantly lower than private ratios (p-value < 0.01). The differences in payment patterns across payer types can inform speculation on the benefits and detriments of the various payment plans available to patients and providers in the US health care system, and in turn shed light on the state of the system overall. From a broad standpoint, our analysis helps to identify trends and pinpoint variations between payers, which can assist those working to improve the equity and efficiency of health care financing in the US.

Third, the relationship between payments and charges for each payer also varies across causes of health care events. When combined with the variation across time and payer, complicated trends emerge. For example, in 1996 public payers paid 54 cents per dollar charged for cardiovascular disease treatment in the inpatient setting. By 2012, this number had dropped to 23 cents per dollar charged. We found less change over time for private and out-of-pocket payers, although the payment ratios decreased during the time of the study. For musculoskeletal disorders, private payers paid 2 fewer cents per dollar charged than public payers in 1996, whereas in 2012 public payers paid less than private by 15 cents per dollar charged.

While more research is necessary to confirm any causal mechanisms underlying our results, we can speculate on what drives the variations across time, payer, and cause. For example, for out-of-pocket payers, we see that the difference between charges and payments is particularly large. Oftentimes these charges are not paid because the uninsured go bankrupt in attempting to cover the entire charge. Out-of-pocket payments, even if only a small fraction of charges, may indicate catastrophic health spending. Research shows this phenomenon to be on the rise in the US, especially for low-income families, the uninsured or underinsured, and those with many long-term conditions [29,30]. In addition, there are some concerns about the increasing popularity of high-deductible health plans, in which the patient is responsible for paying out-of-pocket for a large amount of charges before insurance kicks in. Research investigates whether these plans may incentivize patients to avoid or delay seeking care for fear of incurring debt, which could result in short-term conditions turning into long-term, more expensive ones [31]. In summary, our study shows strikingly low payment ratios for out-of-pocket payers, which supports these pre-existing concerns about the fairness and sustainability of the system regarding out-of-pocket payers.

Past studies have examined how payment ratios have changed over time and found a similar downward trend [8,32]. Our study expands upon previous research in several ways. First, we analyze data in the inpatient setting, which is likely to have a unique medical and financial makeup compared with general hospital data or other departments. Second, we generate nationally representative estimates by incorporating patient weights into our methodological process. Third, we produce credible, gradual time trends over a longer period than previous studies by using a relatively large dataset and running a regression rather than taking averages. Fourth, we generate estimates stratified by primary payer, cause of health care encounter, and year. This level of disaggregation has not been attempted in previous research, and in particular, we produce these cause-specific estimates using policy-relevant GBD categories. Finally, in addition to illuminating trends in payment ratios in the US, our methodology presents a generalizable approach to obtaining nationally representative spending estimates from surveys that only provide information on charges.

Although our study’s findings are consistent with past research and have proven to approximate the NHEA total at an aggregate level (see Fig 4), limitations should be considered. One constraint of this study is the relatively small sample sizes in MEPS data for some causes, and for out-of-pocket payers. These data limitations prevent us from estimating time trends by payer for some causes and motivate us to run the regressions at a less granular GBD cause level than some published GBD estimates [16]. Higher-quality inpatient data containing primary diagnosis, charges, and payments would allow for more granular estimates. Finally, with the data at hand, we are not able to explore variation in the payment ratio across specific medical facilities, geographies, or medical procedures.

## Conclusions

To our knowledge, this study presents the first rigorous investigation of the ratio between payments and charges in the inpatient setting. We find wide variation in these payment ratios across time, payer, and cause. This study provides a model for leveraging smaller datasets with both payment and charge data to obtain spending estimates from larger, more readily available charge datasets. The findings from this study can also serve as a guideline for policymakers working to make health care more transparent and equitable.

## Supporting Information

S1 File Assessing the complex and evolving relationship between charges and payments in US hospitals: 1996–2012—*methods appendix*. (DOCX)
