# Supplementary material for: Assessing the Complex and Evolving Relationship between Charges and Payments in US Hospitals: 1996 – 2012
Source: PLoS One. 2016 Jul 8;11(7):e0157912. doi: 10.1371/journal.pone.0157912 (PMC4938432; doi:10.1371/journal.pone.0157912)
Supplement: S1 File — (DOCX) [file pone.0157912.s001.docx]

# S1 APPENDIX: Assessing the complex and evolving relationship between charges and payments in US hospitals: 1996 – 2012 – *methods appendix*

# Index

Introduction Page 3

Data and data pre-processing Page 3

Charges-to-payments regression Page 5

Application of payment ratios to adjust NIS Page 9

Sensitivity analyses Page 12

# Introduction

This appendix discusses the methodology used to estimate payment-to-charge ratios (payment ratios) in the inpatient setting, and then to apply these findings to adjust a dataset that reports charges only. The datasets used were the Medical Expenditure Panel Survey (MEPS) and the National Inpatient Sample (NIS). Both datasets are produced by the Agency for Healthcare Research and Quality (AHRQ). Both datasets were cleaned and standardized to a common set of cause of healthcare encounter classification, demographic categories, and payer categories. Due to relatively small sample size and the possibility of missing rare causes of healthcare encounters, a moving average approach was used for MEPS, such that data from each year was added to the annual estimates for that year as well as the adjacent years before and after. As a consequence, our 1997 estimates include data from 1996 and 1998, and trends over time are measured to be more gradual. This approach is similar to what is recommended by the AHRQ and methods used by Dunn and colleagues at the US Bureau of Economic Analysis [1]. Both datasets were bootstrapped 1,000 times, using random draws for each observation with replacement. The payment ratio was calculated for each patient. Cause-specific regressions were used to estimate time trends and payment ratios by payer and cause for each of the 1,000 bootstrap draws. These estimated payment ratios are reported in the results section of the main text. A similar regression was performed to generate cause- and year-specific ratios of facility charges over total charges. The resulting facility ratios were multiplied together with the payment ratios to adjust facility charges reported in NIS to reflect total payments. Our primary results are the mean of the 1,000 bootstrap estimates. A 95% uncertainty interval was calculated by assessing the 2.5^th^ and 97.5^th^ percentiles of the 1,000 bootstrap draws.

# Data and data pre-processing

Both MEPS and NIS were processed before running the analysis. International Classification of Disease version 9 (ICD-9) codes, facility charges, facility payments, doctor charges, doctor payments, age, sex, year, and patient weights were pulled from MEPS inpatient files from 1996-2012. ICD-9 codes, facility charges, age, sex, year, and patient weights from 1996-2012 were collected from NIS as well. The two datasets were processed similarly.

### Ages

Patients were placed in five-year age bins starting from five year olds and extending to an open category of those older than 85 years. The 0-4 year old age group was split into a 0-1 bin and a 1-4 bin because of unique inpatient healthcare provided during the first year of life.

### Payers

The data were also categorized by three payer strata: public insurance, private insurance, and people paying out of pocket. NIS has information on the primary payer for each inpatient stay. In MEPS, many inpatient stays reported payments from multiple payers, reflecting co-payments, deductibles, or the purchase of Medicare Supplement Insurance. When multiple payers were listed, the payer who covered the largest portion of the payment was designated as the primary payer, and the entire payment was attributed to this payer. For example, if Medicare paid 75% of a patient’s total payment and the other 25% was out-of-pocket, the observation was assigned to the “public” stratum. As seen in Fig S1, the primary payer often covered the majority of each payment.

**Fig S1.** Proportions of the total payment paid by the primary payer in MEPS.


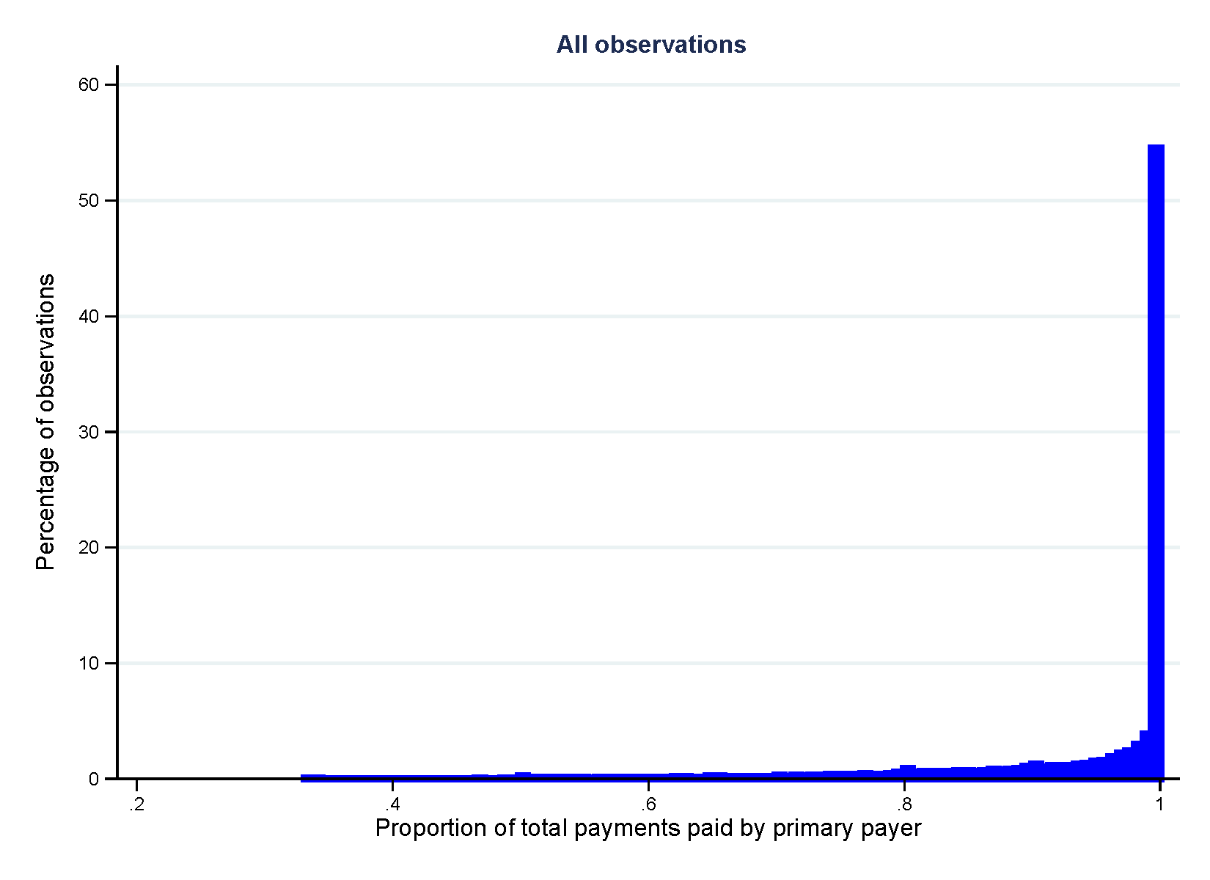


### Diagnoses

While NIS reports a primary diagnosis, MEPS does not. For MEPS, the primary diagnosis was assigned to be the first listed ICD-9 code. The causes associated with payment ratios in our analysis were then based upon this primary diagnosis. For both datasets, we used the Global Burden of Disease 2013 study (GBD 2013) as our underlying framework for disease classification [2,3]. This cause framework aggregates the 17,849 ICD-9 codes into 289 distinct causes according to clinically relevant groupings of codes [4]. Within the GBD framework, causes can be further aggregated into less granular classifications depending on the policy purpose. In this study we also included an “expenditure-only” category of causes, which tracks encounters with the health system that are not associated with disease burden, and are therefore excluded from GBD. Examples are organ donation, and healthy pregnancy and postpartum care. We mapped the primary diagnoses found in MEPS and NIS to 32 unique causes of healthcare encounters.

Both datasets contained GBD “garbage codes,” defined as causes that are vague or do not report the underlying causes of healthcare encounters [5]. In addition, diagnoses were reset to garbage if they broke plausible age or sex restrictions, such as Alzheimer’s disease in five year olds, or testicular cancer in women. These observations could not be included in the analysis as it was unclear what was being treated during these healthcare events. Therefore, these patient-level records were removed from both datasets. This decision was based on the assumption that the distribution of payment ratios in each valid GBD cause would not systematically change if the observations prescribed to ICD-9 codes that map to garbage were probabilistically assigned ICD-9 codes that mapped to valid GBD causes. Under this assumption, dropping these data does not bias the results.

For privacy reasons, MEPS truncates ICD-9 codes to three digits. In some cases the truncated three-digit ICD-9 codes map to a different GBD cause than the full four- or five-digit code. This could lead to misclassification of a portion of the encounters. For some GBD causes, such as atrial fibrillation and eating disorders, only full four- and five-digit codes map to these causes while the truncated code maps to something else. This means that these GBD causes cannot appear in the original MEPS data. To make MEPS diagnoses comparable with NIS diagnoses, which are coded to the full five-digit form, a probabilistic assignment was performed. The conditional probabilities used in this assignment were calculated using full four- or five-digit codes from NIS. Because this process was executed separately for each bootstrap draw, it resulted in an increase in the number of observations in the dataset. For one draw, an observation may be assigned one five-digit ICD-9 code. For a separate draw, this same observation may be assigned a different five-digit ICD-9 code, thus increasing the apparent size of the dataset.

ICD-9 codes injuries using two distinct classifications: N-codes and E-codes. N-codes describe the “nature” of the injury, and represent a physician’s diagnosis; e.g. the patient has a joint dislocation or a hip fracture. In contrast, E-codes describe the “external” cause of injury, e.g. an automobile accident. The GBD classification system relies on E-codes, as they are more easily connected to prevention measures and therefore more policy relevant. In order to ensure that spending on the treatment of injuries was attributed to GBD causes, an adjustment was applied to observations with N-code as primary diagnosis. The methods used for the injury adjustment were based off previous work [6]. In both MEPS and NIS, E-codes were filled in for observations that had only an N-code reported. First, injury maps were created from NIS data. The maps contain the conditional probability of a patient having a particular E-code, given that they were assigned a specific N-code. The most detailed version contains probabilities that are age and sex specific, the middle version is only age specific, and the least detailed version has generic probabilities using all N-code and E-code combinations found in NIS. Cycling through these maps from most to least detailed, each observation with an N-code but no E-code was assigned an E-code according to these conditional probabilities.

### Years

Due to relatively small sample size and the possibility of missing rare causes of healthcare encounters, a moving average approach was used for MEPS, such that data from each year was added to the annual estimates for that year as well as the adjacent years before and after. As a consequence, our 1997 estimates include data from 1996 and 1998, and trends over time are measured to be more gradual. This approach is similar to what is recommended by the AHRQ and methods used by Dunn and colleagues at the US Bureau of Economic Analysis [1].

# Charges-to-payments regression

Payments were divided by charges to produce patient-level payment ratios for analysis. Observations in which payments were greater than charges (2.2% of total) were considered to be errors, and charges were re-coded to be equal to payments. To test this assumption, we ran a sensitivity analysis, described in the fifth section of this appendix. Throughout this analysis we stratify the payment ratio by payer, cause, and year, but not by age or sex. We explored all of these demographics as possible variables and found that the payment ratio does not vary systematically across age or sex. For sex, the average payment ratio was calculated for every age, sex, year, payer, and cause combination. Averages that had the same age, year, payer, and cause but different sex were then paired. The difference between the matched payment ratios was taken for each pairing, and Fig S2 shows a histogram of these values. The symmetrical distribution of these differences suggests that sex is unimportant in determining payment ratios. For age, we examined the payment ratios for public payer plotted against age for three causes. Fig S3 shows there is no apparent trend in the payment ratio across the age profile. Similar plots assess systematic variation across the age profile for other causes and payers confirm this observation.

**Fig S2. Distribution of differences between paired male and female payment ratios.**

**
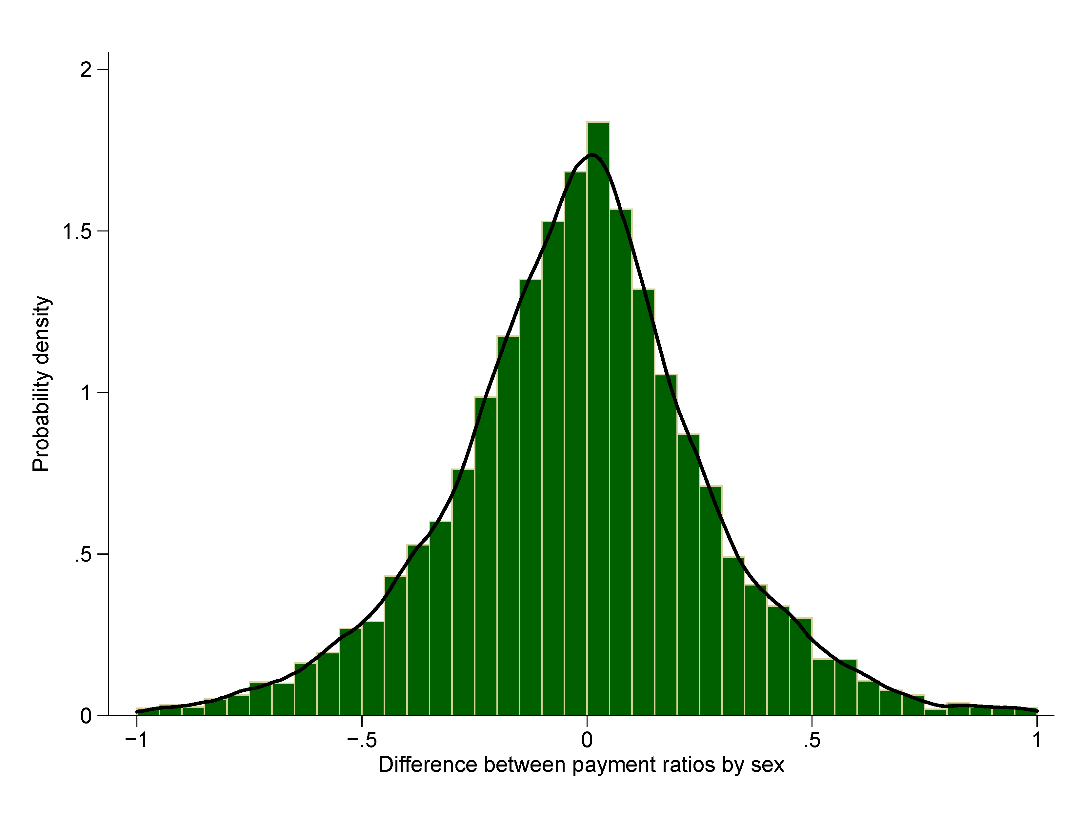
**

**Fig S3. Public payer payment ratios for three causes across the age profile.**


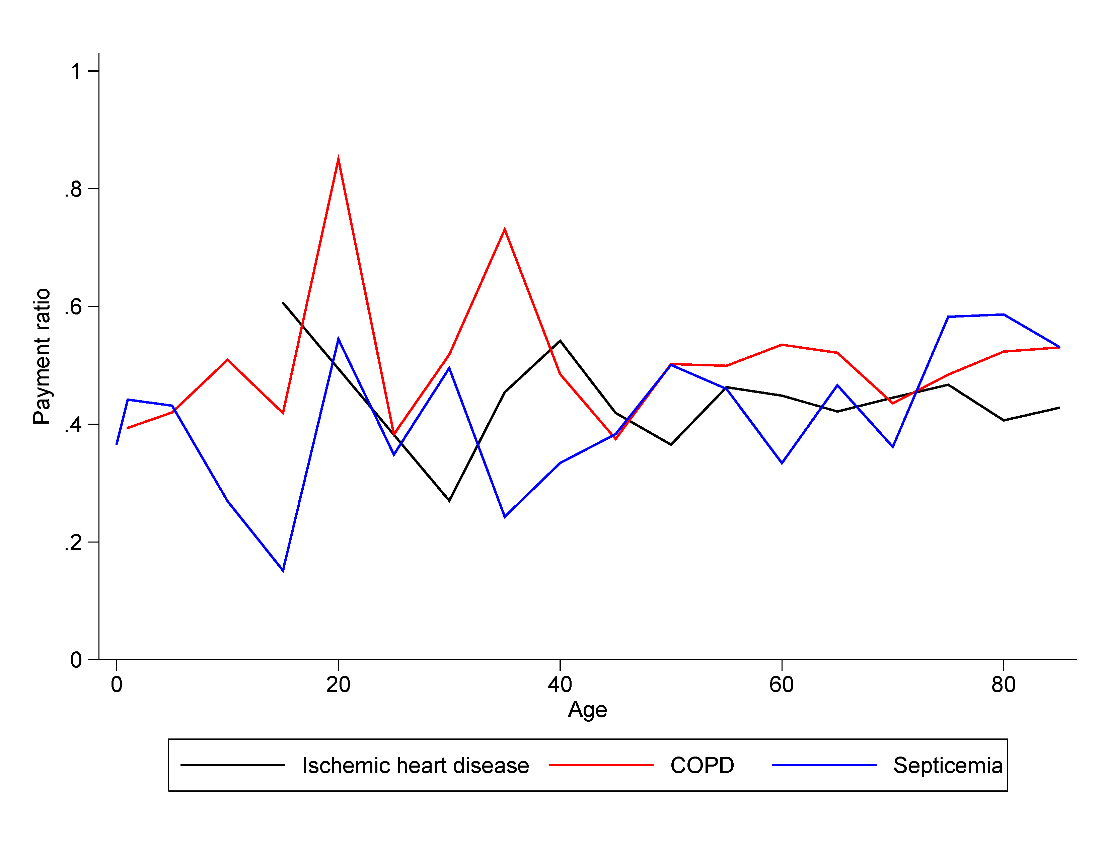


A cause- and draw- specific linear regression model was estimated at the patient level. The payment ratio was regressed on payer indicators and the interaction between the payer indicators and time (measured in years). For each cause, the equation was as follows:

Equation (1):

$$\left( \frac{payments}{charges} \right)_{i}= \beta_{0}\cdot{public}_{i}+ \beta_{1}\cdot{private}_{i}+ \beta_{2}\cdot{oop}_{i}+ \beta_{3}\cdot{public}_{i}\cdot year+ \beta_{4}\cdot{private}_{i}\cdot year+ \beta_{5}\cdot{oop}_{i}\cdot year+ \varepsilon_{i}$$

The product of patient weight and patient-level charge was used as a frequency weight in the regression. These custom weights allowed the regression to be nationally representative by adjusting for two issues. First, the patient weights from MEPS addressed the survey’s sampling frame. Using these weights makes the results representative of the US population. Second, incorporating charges into the regression weights means that larger charges are up-weighed. By inspection, the size of the charge itself influenced payment ratios such that the larger a charge, the smaller the proportion a payer was likely to pay. However, observations with charges larger than the mean for a given cause, year, and payer are relatively small in number. If we weight by charges, these payment ratios with high charges count more. As these high charge payment ratios tend to be low, incorporating charge into the regression weight pushes our estimates downward. Thus if we didn’t weight by them our ratios would be biased upward, and our results would not accurately describe the total amount paid divided by the total amount charged in the inpatient setting. These custom weights were incorporated as frequency weights in the regression. In other words, if a weight of 1,000 was attributed to an observation, the regression treated the observation as if it were 1,000 distinct data points. Under this conceptualization, a charge of $100 with a ratio of 0.80 would be equivalent to a ratio of 0.80 for 100 separate $1 charges. By definition, a frequency weight of 100 is treated as if an observation occurred 100 times, so this weighting choice is valid.

Fig S4 shows payment ratios taken from 2012 data for publically insured patients being treated for cardiovascular diseases. These ratios were plotted against the total amount charged. The horizontal line is the average payment ratio for all points shown. The downward trend of the dots shows that there is a systematic bias toward lower payment ratios for visits with larger total charges.

**Fig S4.** **Patient-level payment ratios from MEPS over charges from MEPS.**


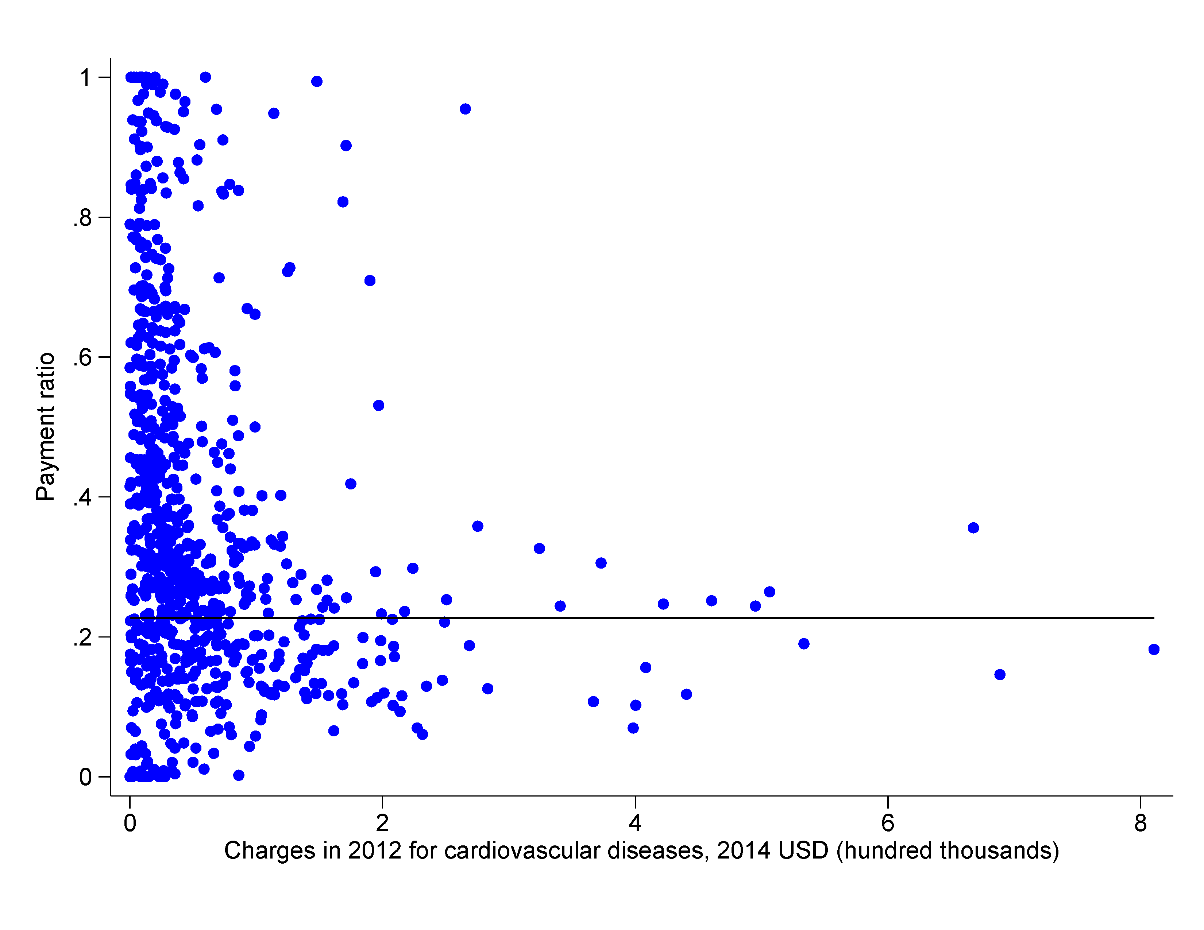


For a given cause and draw combination, equation (1) was used for all payers with more than 200 observations. This threshold was not met for 40,044 of our 96,000 cause, strata, and bootstrap draw combinations. Out of these instances, 19,149 were for OOP payers. When a cause, draw, and payer combination did not meet this threshold, the corresponding payer-year interaction term was dropped. Table S1 lists how many draws passed this threshold of 200 observations, by payer, and cause.

**Table S1:** Number of draws with at least 200 observations for each cause and payer.

| **Cause** | **Public** | **Private** | **Out-of-pocket** |
| --- | --- | --- | --- |
| HIV/AIDS & tuberculosis | 0 | 991 | 0 |
| Diarrhea/LRI/other | 1000 | 1000 | 175 |
| NTDs & malaria | 0 | 0 | 0 |
| Maternal disorders | 1000 | 1000 | 0 |
| Neonatal disorders | 0 | 50 | 0 |
| Nutritional deficiencies | 393 | 1000 | 0 |
| Other communicable, maternal, neonatal, and nutritional diseases | 1000 | 1000 | 0 |
| Communicable NEC* | 0 | 0 | 0 |
| Neoplasms | 1000 | 1000 | 997 |
| Other non-communicable | 1000 | 1000 | 1000 |
| Cardiovascular diseases | 1000 | 1000 | 1000 |
| Chronic respiratory | 1000 | 1000 | 861 |
| Cirrhosis | 35 | 1000 | 0 |
| Digestive diseases | 1000 | 1000 | 1000 |
| Neurological disorders | 1000 | 1000 | 0 |
| Mental & substance use | 1000 | 1000 | 1000 |
| Diabetes/urog/blood/endo | 1000 | 1000 | 1000 |
| Musculoskeletal disorders | 1000 | 1000 | 0 |
| Transport injuries | 1000 | 1000 | 0 |
| Unintentional injuries | 1000 | 1000 | 1000 |
| Intentional injuries | 1000 | 1000 | 0 |
| War & disaster | 0 | 0 | 0 |
| Injuries NEC* | 0 | 0 | 0 |
| Well person | 1000 | 1000 | 1000 |
| Pregnancy and postpartum care | 1000 | 1000 | 1000 |
| Dental | 1000 | 1000 | 1000 |
| Family planning | 0 | 0 | 0 |
| Donor | 998 | 998 | 998 |
| Counselling services | 0 | 0 | 0 |
| Clinical support | 0 | 0 | 0 |
| Social services | 820 | 820 | 820 |
| *NEC=Not Elsewhere Classified |  |  |  |

The first version of the model supposes that the payment ratio changes over time for that payer, while the second version simply takes the average over the entire time period. Regarding the threshold chosen for this distinction, there are conflicting opinions concerning the number of observations needed to run a multivariate linear regression [7,8]. The threshold used was a conservative estimate, motivated both by the consideration that estimating no change in payment ratio over time is a better option than estimating a spurious trend biased by data outliers, and by the fact that data points were borrowed from neighboring years in MEPS, so the underlying source of data has fewer observations than the dataset that is regressed.

# Application of payment ratios to adjust NIS

An additional regression was needed to apply our estimated payment ratios to NIS. Hospital charges are often split into two components: facility charges and professional charges. MEPS reports both types of charges, whereas NIS reports facility charges only. This paper addresses the cost of receiving inpatient care from the perspective of the patient, so total charges and total payments are the metrics of interest. These totals are equivalent to the sum of facility charges and professional charges, or the sum of facility payments and professional payments, respectively. The payment to charge regression detailed above estimates the ratio of *total* payments over *total* charges. Therefore, a facility charge to total charge conversion was needed in order to estimate the total payments in NIS. This second conversion follows a similar form as the first and can be found in equation (2). The accuracy of the second model is limited by differences in how the two data sources define facility charges. MEPS defines facility charges as the amount a hospital charges a patient. This number often includes fees for a physician’s work. However, some physicians charge separately, and these separate charges are labeled as professional charges [9]. NIS, however, separates all hospital charges from physician charges when possible [10]. Consequently, we may overestimate the ratio of facility charge to total charge.

Equation (2):

$\left( \frac{total charges}{facility charnges} \right)_{i}= \beta_{0}+ \beta_{1}{year}_{i}+\varepsilon_{i}$

The ratio of facility charges to total charges was the dependent variable in this second regression. This ratio was considered a function of cause and time. By inspection, this ratio was unrelated to age, sex, and payer. Further, charges are widely known to be independent of payer, as all charges come from a hospital’s charge master [11]. The regression given by equation (2) was run for each draw for each cause, with patient-weighted total charges as the regression weight. Finally, a facility-charge to total-payment conversion factor was calculated for each cause and payer by combining the resulting ratios from equations (1) and (2), as shown in equation (3).

Equation (3):

conversion factor = $\left( \frac{total charges}{facility charges} \right)_{i}\left( \frac{total payment}{total charges} \right)_{i}$

Because of the payer-specific payment ratio on the right-hand side, Equation (3) is specific to year, cause, and payer. However, the NIS charge estimates we adjusted were not stratified by payer. To be able to multiply the conversion factors into NIS and obtain expenditure estimates, a weighted average across payer was taken in order to obtain a single conversion factor for each cause, year, and draw combination. The weights for the average were calculated for each draw by taking the cause-specific proportions of facility charges for each payer at GBD cause level three from NIS. In doing this, we take into account the different distribution of payers across cause. For example, cardiovascular diseases are more common among the elderly population, and consequently Medicare often is the primary payer for treating these diseases. Thus when calculating the conversion factor, the method used gave more weight to the public payment ratio for cardiovascular diseases. While the main analysis was run for the 32 causes at cause level two in order to increase statistical power, weights for the weighted average were taken at the more granular level, GBD cause level three, which contains 187 cause categories. This decision was made because NIS is large enough to provide meaningful charge information when charges are stratified by level-three cause, and year. The weighted average of payment ratios across payer resulted in the final conversion factor, which was applied to NIS by year and level-three cause.

This methodology was tested in-sample by applying the conversion factors to MEPS facility charges. The accuracy of the method can be checked by comparing the values predicted by the model to the actual observed values. These results are shown in Fig S5. The predicted payments and reported payments are closest together in the middle of the time series and diverge toward the beginning and end of the period of interest. This finding is expected, considering the payment ratio was modeled with a linear regression, while the true time trend is likely not completely linear. Nevertheless, the two lines are highly overlapping, suggesting that the linear model and the calculated conversion factors are a valid adjustment.

**Fig S5.** **Reported payments and charges from MEPS over time, compared with payments predicted by applying results of model to MEPS charge data.**


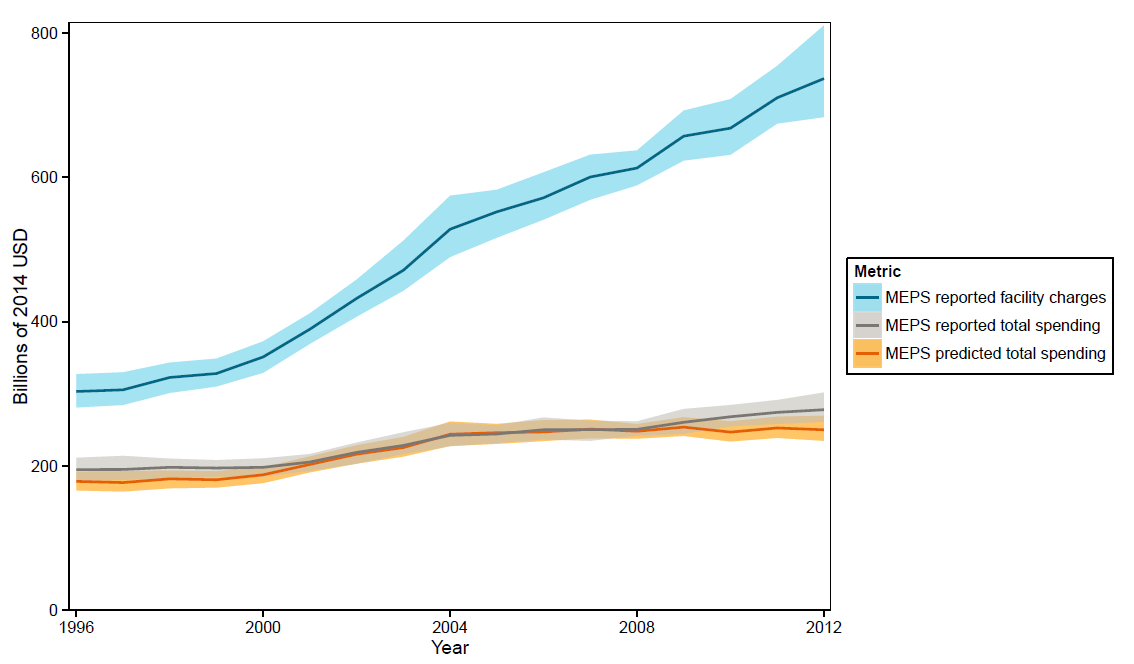


# Adjusting National Health Expenditure Account estimate for comparison

To test the validity of our model, we compared our adjusted NIS payments to community hospital inpatient spending estimates derived from the National Health Expenditure Account (NHEA). The Centers for Medicare & Medicaid Services publish annual estimates of health spending in the US by type of good or service delivered. As none of these types of service aligned perfectly with inpatient care, we adjusted the NHEA spending estimates to align with the sampling frame of NIS.

We first applied previously published adjustments to correct the NHEA hospital spending estimates [12, 13]. Specifically, the NHEA hospital spending estimates include spending by entities that are excluded from NIS, such as hospital-owned nursing facilities, or hospital-run home health care. We next multiplied this adjusted hospital-spending estimate by the fraction of hospital spending that occurred in non-federal, community hospitals. This fraction was taken from research by the American Hospital Association, and was from 2012 [14]. Information comparing community hospital spending to all hospital spending was only available for 2012, 2013, and 2014. Since the fraction was relatively constant for all three of these years, the 2012 number was taken to represent the fraction from 1996 to 2012. Next, we removed hospital spending on emergency and outpatient departments. To do this we used payments from MEPS inpatient, hospital outpatient and emergency department files for each year to calculate the proportion of hospital spending which is from inpatient admissions. This fraction was applied to the adjusted NHEA estimate. The result represents total US spending on community hospital admissions and matches the sampling frame of NIS. Finally, we subtracted out the amount spent on inpatient stays attributed to GBD “garbage codes” that was present in MEPS.

# Sensitivity analyses

We ran three sensitivity analyses to test whether altering the assumptions in our model made a significant difference in our results. Fig S6 depicts the results of the three sensitivity analyses against those of the final methods to facilitate comparison between methods.

**Fig S6.** Estimated payments over time using each of three sensitivity analyses and the final method.


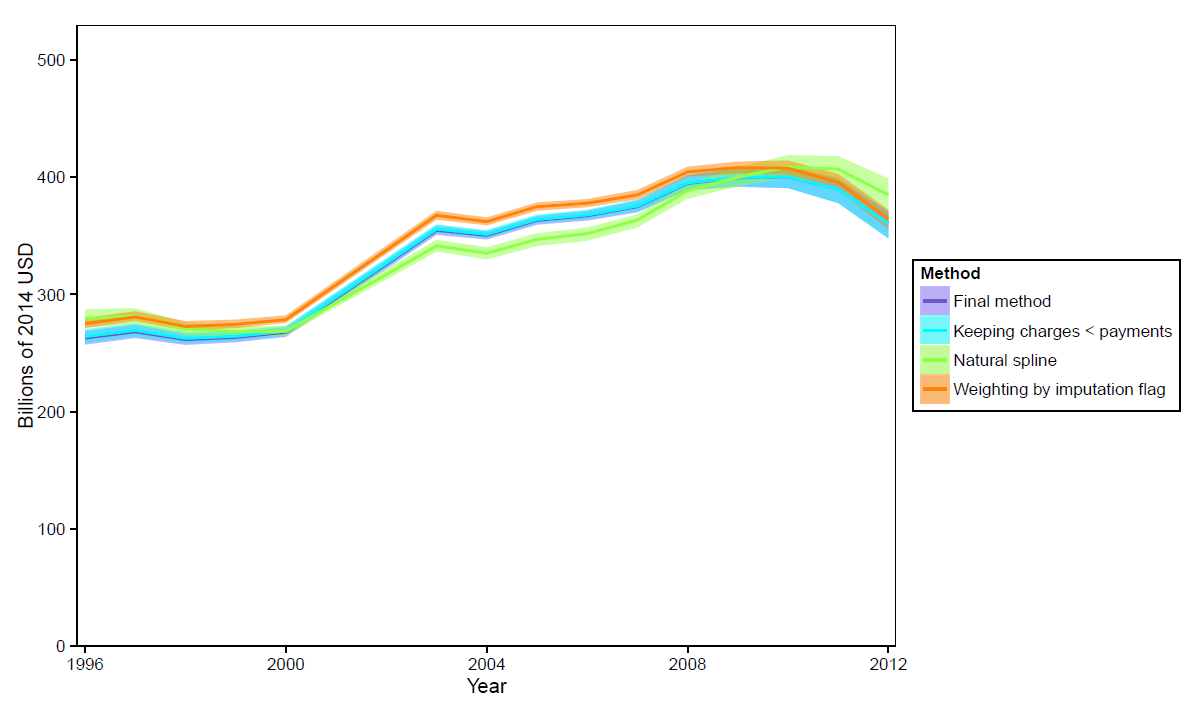


AHRQ provides MEPS data in a processed form. Part of this processing involves imputation of missing payment and charge data. Nonrandom measurement error would bias this paper’s estimates, so a sensitivity analysis was run in which the payment ratio regression was weighted by an imputation index. The imputation index is provided by MEPS, and takes on five values, ranging from complete payment and charge information provided by a medical provider, to fully imputed [9]. The values were ordered so that the lowest value indicated the most amount of imputation. This indicator was used as an importance weight. To continue to take into account the relationship between larger charges and lower payment ratios, observations were duplicated. The number of duplicates created was the ceiling of the quotient: the amount charged for a patient divided by the median of the charges in the dataset. In other words, whereas the final method used in this paper was to weight the regression by patient weight and charge amount, this sensitivity analysis weighted the regression by an imputation flag and charge amount. As seen in Fig S6, this alternative weighting system for the regression did not substantially change our results.

A second sensitivity analysis was performed to test the assumption that a cause-specific payment ratio has a linear relationship with time. In this analysis, a natural spline regression was run instead of a linear regression. This regression was run with the same weights used in the final analysis. Fig S6 also shows that modeling a nonlinear relationship between payment ratio and time did not substantially alter the results.

Finally, there were a small number of observations in which the reported charges were less than the payments. These were considered to be errors, and in the main analysis we reset charges to be equal to the corresponding payments, creating a payment ratio of one for these observations. The decision to set charges down to payments rather than increasing payments up to charges meant that these observations would also be slightly down-weighted, since charges were incorporated into the regression weights. To test this procedure we ran the regression without resetting charges in these observations. Fig S6 shows that this adjustment did not substantially change our results.

# References

1. Sommers J. An Examination of State Estimates Using Multiple Years of Data from the Medical Expenditure Panel Survey, Household Component. Agency Healthc Res Qual. 2006;Working Paper. Available: http://meps.ahrq.gov/mepsweb/data_files/publications/workingpapers/wp_06004.pdf
2. GBD 2013 Mortality and Causes of Death Collaborators. Global, regional, and national age-sex specific all-cause and cause-specific mortality for 240 causes of death, 1990-2013: a systematic analysis for the Global Burden of Disease Study 2013. Lancet Lond Engl. 2015;385: 117–171. doi:10.1016/S0140-6736(14)61682-2
3. Global Burden of Disease Study 2013 Collaborators. Global, regional, and national incidence, prevalence, and years lived with disability for 301 acute and chronic diseases and injuries in 188 countries, 1990-2013: a systematic analysis for the Global Burden of Disease Study 2013. Lancet Lond Engl. 2015; doi:10.1016/S0140-6736(15)60692-4
4. ICD - ICD-10-CM - International Classification of Diseases,(ICD-10-CM/PCS Transition [Internet]. [cited 28 Jul 2015]. Available: http://www.cdc.gov/nchs/icd/icd10cm_pcs_background.htm
5. Lopez A, Mathers C, Ezzati M, Jamison D, Murray C. The Burden of Disease and Mortality by Condition: Data, Methods, and Results for 2001. Global Burden of Disease and Risk Factors. Available: http://dcp-3.org/sites/default/files/gbd/GBD03.pdf
6. Vos T, Flaxman AD, Naghavi M, Lozano R, Michaud C, Ezzati M, et al. Years lived with disability (YLDs) for 1160 sequelae of 289 diseases and injuries 1990-2010: a systematic analysis for the Global Burden of Disease Study 2010. Lancet Lond Engl. 2012;380: 2163–2196. doi:10.1016/S0140-6736(12)61729-2
7. Schneider A, Hommel G, Blettner M. Linear Regression Analysis. Dtsch Ärzteblatt Int. 2010;107: 776–782. doi:10.3238/arztebl.2010.0776
8. Green SB. How Many Subjects Does It Take To Do A Regression Analysis. Multivar Behav Res. 1991;26: 499–510. doi:10.1207/s15327906mbr2603_7
9. MEPS HC135D: 2010 Hospital Inpatient Stays. Agency for Healthcare Research and Quality; 2012.
10. HCUP NIS Desption of Data Elements [Internet]. Healthcare Cost and Utilization Project; September 08. Available: www.hcup-us.ahrq.gov/db/vars/totchg/nisnote.jsp
11. Reinhardt UE. The Pricing Of U.S. Hospital Services: Chaos Behind A Veil Of Secrecy. Health Aff (Millwood). 2006;25: 57–69. doi:10.1377/hlthaff.25.1.57
12. Roehrig C, Miller G, Lake C, Bryant J. National Health Spending By Medical Condition, 1996–2005. Health Aff (Millwood). 2009;28: w358–w367. doi:10.1377/hlthaff.28.2.w358
13. Bernard D, Cowan C, Selden T, Cai L, Catlin A, Heffler S. Reconciling Medical Expenditure Estimates from the MEPS and NHEA, 2007. Medicare Medicaid Res Rev. 2012;2. doi:10.5600/mmrr.002.04.a09
14. "Fast Facts On US Hospitals". *Aha.org*. N.p., 2016. Web. 22 Apr. 2016.
